# A Phase-Field Study of Microstructure Evolution in Tungsten Polycrystalline under He/D Irradiation

**DOI:** 10.3390/ma14237433

**Published:** 2021-12-03

**Authors:** You-Sung Han

**Affiliations:** Department of Mechatronics Engineering, Incheon National University, 119 Academy-ro, Yeonsu-gu, Incheon 22012, Korea; yshan@inu.ac.kr

**Keywords:** microstructure evolution, grain boundary, phase-field modeling, void growth, dislocation

## Abstract

Analyses in the present study focus on understanding the evolution of the tungsten microstructure under He/D irradiation. A fractal dimension analysis was utilized to characterize the structural pattern of the microstructure irradiated by both low (10–80 eV) and high (8–30 keV) irradiation energy. All examined W microstructures show a direct correlation between the fractal dimension and irradiation energy. Analyses establish an empirical relation expressing a change in the microstructure as a function of the irradiation energy based on the changes in the fractal dimension of the microstructures. The proposed relation was implemented in the phase-field model formulation with an account of the interfacial energy induced by the crystallographic mismatch between grains under irradiation. The current phase-field model captures the evolution of the void under irradiation, including nucleation and the growth of voids, and sink efficiency for vacancy annihilation in the vicinity of grain boundaries.

## 1. Introduction

Tungsten (W) has excellent material properties at high temperatures. W has the highest melting point among metals. It also has a high threshold for physical sputtering energy (E_th_ ≅ 200 eV for deuterium) and is thus known as ‘low erosion’ material [1]. In addition, W has high thermal conductivity and good thermal shock resistance. Because of such excellent properties at high temperatures, W is one of the promising candidate materials for structural components of nuclear fusion reactors.

The structural components in nuclear power systems are exposed to high levels of irradiation energy, heat flux, and thermo-mechanical stresses. Materials in a nuclear reactor undergo several phenomena such as defect formation, the interaction of defects with impurities, void migration, and defect recovery. A high temperature gradient in a nuclear reactor leads to the migration of small voids and their coalescence into larger voids. Kinetics of voids’ evolution such as their volume fraction, morphology, and size could significantly affect the material properties, including fracture toughness, tensile strength, thermal conductivity, and so on. Therefore, such effects should be taken into account for a more precise understanding of the material’s behaviors in nuclear reactor environments.

Plasma-facing components (e.g., first wall and divertor) for next-generation nuclear fusion reactors are expected to be exposed to irradiation of over 100 displacement per atom (dpa) [2]. In such a high-irradiation environment, the void-induced swelling leads to microstructural change and thus the deterioration of material properties. The void evolution (nucleation, growth, and aggregation) during irradiation has been observed in several materials [3,4,5,6,7,8]. The porosity volume fraction, morphology, and kinetics of voids significantly affect material properties [9]. Therefore, it is important to understand and predict the evolution of the microstructure under an irradiation environment to ensure the reliability of the nuclear reactor system.

Microstructure evolution under irradiation involves complex processes, including diffusion, interaction, clustering, and thermal recovery of voids. Point defects produced by displacement cascades interact with each other, aggregate into clusters, and thus form voids/bubbles. Some of these point defects are annealed at high temperatures when the energy of the material system exceeds its activation energy for the recombination. These processes can only be captured at the mesoscopic length and time scales.

Phase-field simulations have been performed to study the phase transformation and the kinetics of microstructural features in a variety of materials processes, including crack propagation [10,11], phase transformations [12,13], grain evolution [14], and so on. In the phase-field model, the phase-field variables, which are continuous across interfacial regions, are employed to represent the microstructure. They can be composition, phases, grain orientation, or other microstructural features. In the phase-field model, the microstructure is evolved by the Cahn–Hilliard diffusion equation [15], which uses thermodynamic free energy [16] obtained from experiments, atomistic calculations, and thermodynamic calculations. The phase-field variables evolve such that the total free energy of the material system reduces.

A variety of experimental studies on polycrystalline W has been conducted to examine the evolution of its microstructural features under irradiation. However, the exact mechanism of microstructure evolution as a function of irradiation remains unclear. This issue becomes more complicated by the fact that it is not only challenging but also costly to perform the experiments with a realistic level of irradiation energy and irradiation exposure time. For this reason, most studies analyze the microstructure irradiated with a low or small range of irradiation energies.

The phase-field simulations have been conducted to study the change of microstructural features under irradiation, such as nucleation and the growth of gas bubbles and voids, and the evolution of the precipitate morphology [17,18,19,20,21]. However, the change in grain morphology and interfacial energy induced by the crystallographic mismatch between grains under irradiation have not been explicitly taken into account in such works. Han and Tomar [22] used a fractal-dimension-based approach to correlate the changes in the atomic configuration of W grain boundaries (GBs) with the corresponding fractal dimensions. It was shown that the bond strength of the examined W GBs is directly correlated with the fractal dimension of the corresponding nanostructures.

In the present work, the fractal dimensions of irradiated W microstructures are analyzed for a wide range of He/D irradiation energy. Analyses establish an empirical relation expressing change in microstructure as a function of the microstructural fractal dimension. In order to study the evolution of voids in W polycrystalline under He/D irradiation, the proposed relation was implemented in the phase-field model formulation with an account of the interfacial energy induced by the grain mismatch under He/D irradiation. The remainder of this paper is organized as follows. Details on the free energy of the system and kinetic equation for the phase-field formulation are provided in Section 2. The evolution of microstructural features in W polycrystalline is described in Section 3. Then, the effect of He/D irradiation energy and temperature on the evolution of voids in W polycrystalline is discussed in Section 4. Finally, concluding remarks are presented in Section 5.

## 2. Materials and Methods

### 2.1. The Calculation of Fractal Dimension

The fractal dimensions of a range of W microstructures (available in the literature) He/D irradiated by both low energy (10–80 eV) and high energy (8–30 keV) were calculated using the box-counting method. In this work, the program developed by Moisy [23] was used for the fractal dimension calculation. The images of the irradiated W microstructures from the previous scanning electron microscope (SEM) and transmission electron microscope (TEM) studies were digitized and prepared for fractal analyses in order to investigate the correlation of fractal dimension of the microstructures with He/D irradiation energy [24,25,26,27,28,29,30,31,32,33,34]. The digitized images of the examined W microstructures were imported into the fractal analysis program, and then the fractal dimension was calculated using the box-counting method. Each digitalized image of W microstructures had a size of 420 × 315 pixels. As Dathe and Baveye [35] reported, the change of the magnification of the SEM/TEM images could affect the values of the surface fractal dimension. If one zooms in for smaller details of a structure, then the value of the fractal dimension calculated using the box-counting method will decrease accordingly. It is worth noting that the microscopic images with the same order of magnification were used in the present study to reduce the influence of image magnification on the surface fractal dimension. Details on the box-counting method, fractal dimension calculation, and validation of the fractal analysis program can be found in Han and Tomar [22].

**Figure 1:**
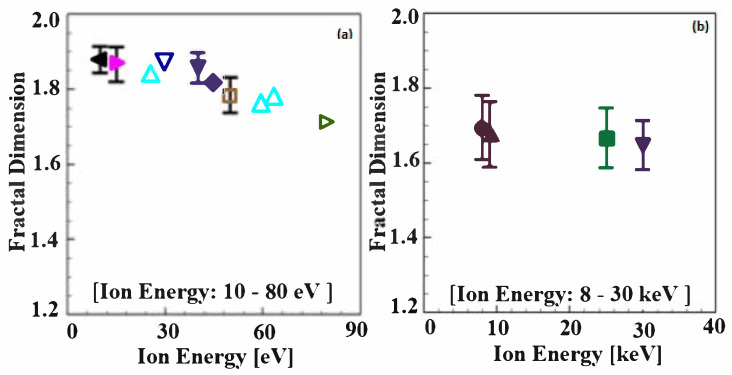
Fractal dimension of irradiated W microstructures as a function of He/D irradiation ion energy: (**a**) Low ion energy (10–80 eV); (**b**) high ion energy (8–30 keV). Details on irradiation conditions and the references are listed in Table 1.

Figure 1 shows the fractal dimension of the examined irradiated W microstructure as a function of He/D irradiation energy. The reference sources and irradiation conditions for the microstructures are listed in Table 1. Note that the *x*-axis scales in Figure 1a,b are eV and keV, respectively. In Figure 1a, the mean fractal dimension of W microstructures irradiated by 10 eV energy is calculated as 1.878 and the value of fractal dimension decreases to 1.716 for the microstructures irradiated by 80 eV energy. While the values of the fractal dimension slightly increase with the increase in irradiation energy from 26 eV to 30 eV, the fractal dimension of the irradiated W microstructures overall decreases as the irradiation energy changes from E = 10 eV to E = 80 eV. In the case of high levels of irradiation, there are only four available microscopic studies on irradiated W microstructures that provide images with high enough resolution to allow for the fractal analysis [30,31,32,34]. The microscopic images from those studies were used for the fractal dimension calculations in the present study (Figure 1b). In the case of the W microstructure irradiated by irradiation energy from E = 8 keV to 30 keV, the fractal dimensions were found to be in the range of 1.583 to 1.764. The overall trend is a reduction in the fractal dimension with an increase in He/D irradiation exposure. The mean fractal dimensions of the microstructures in Figure 1b are calculated as 1.707, 1.677, 1.668, and 1.658 for E = 8 keV, 9 keV, 25 keV, and 30 keV, respectively. It is worth noting that, overall, in Figure 1, the fractal dimensions of the irradiated W microstructures decrease with the increase in irradiation energy. In addition, the sensitivity of the fractal dimension to the change in the irradiation energy becomes small for higher levels of the irradiation energy.

Based on the linear relationship between the fractal dimension and the He/D irradiation energy shown in Figure 1, an empirical relationship expressing the fractal dimension of the examined W microstructures as a function of the irradiation energy can be developed.

In the initial work, the effect of the irradiation exposure time on the fractal dimension of the W microstructures was also investigated. Such information was only available for low irradiation energy. In addition, the SEM/TEM images of the W microstructures irradiated by the same level of irradiation energy with a different irradiation exposure time are required for the correct investigation. However, there are only two available microscopic studies (*E* = 15 eV, 50 eV) on the irradiated W microstructures that satisfy such conditions and provide images with high enough resolution to allow for fractal analysis.

**Figure 2:**
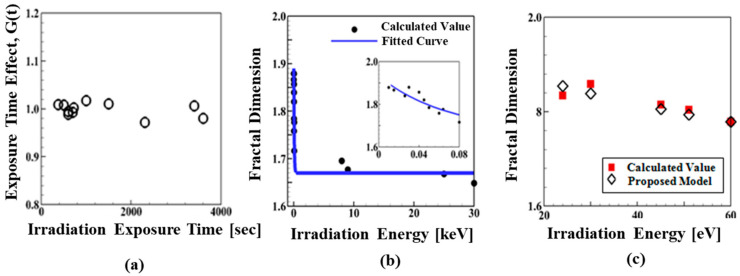
(**a**) The influence of irradiation exposure time on the fractal dimension of the examined W microstructures (See the text for details). (**b**) Fractal dimension evolution as a function of irradiation energy. (**c**) A comparison of the calculated values with the predictions from the proposed relation of fractal dimension of the irradiated W microstructures. Microscopic images in ([24,26,28,29,30,31,32,33]) are used for the comparison.

It was initially assumed that the fractal dimension (*D*) of the irradiated W microstructures is calculated by the product of functions of irradiation energy (*E*) and irradiation exposure time (*t*) as *D* = *f*(*E*)*G*(*t*). However, the analysis showed that the irradiation exposure time led to the a in the fractal dimension of the microstructures only up to 5%, as shown in Figure 2b. Noting the previous studies reporting the minimum and average displacement threshold energy, *E_d_* of tungsten as 68 eV [36] and 90 eV [37], respectively. The reason for such an insignificant effect is that most of the microscopic images used were obtained from the irradiation experiments with low irradiation energy (less or slightly higher than displacement threshold energy) and short irradiation exposure time due to the limitation of available experiment data. Therefore, in order to derive the relation, it is assumed that the fractal dimension of the irradiated W microstructures can be calculated as a function of the irradiation energy as follows:

(1) $$D=f\left(E\right)$$

where D is the fractal dimension of the examined W microstructure; E is the level of the irradiation energy. Because the fractal dimensions of the microstructures decrease with an increase in irradiation energy, and the sensitivity of the fractal dimension to the change in irradiation energy reduces at high levels of irradiation energy, the function $f\left(E\right)$ is chosen to be an exponential function. Using the nonlinear least-squares method, curve-fitting was performed and the function *f*(*E*) in Equation (1) was obtained as

(2) $$D=f\left(E\right)=\exp\left(a_{1}E/a_{2}\right)+a_{3}$$

where $a_{1}$ = 0.262, $a_{2}$ = −0.068, and $a_{3}$ = 1.670. The calculated values and fitting results of the fractal dimension are compared in Figure 2a as a function of the irradiation energy. For better illustration, those values for low irradiation energy (up to 0.08 keV) are plotted in the inset of Figure 2a. For the purpose of verification of the relation, the predictions from the proposed relation are compared with the fractal dimension calculations, which were not included in the empirical relation development. Microscopic images in [24,26,28,33] are used for the comparison. As shown in Figure 2c, the predictions from the proposed relation are, overall, in good agreement with the fractal dimension calculations of the microstructures.

The examined microstructures in the present study represent a small portion of an actual irradiated microstructure. Significant variations of microstructures are possible depending on irradiation conditions. However, it is not only challenging but also costly to perform the irradiation experiments with an account of realistic levels of irradiation energy and irradiation exposure time. For these reasons, the limited data are only available for the microstructures irradiated under a nuclear irradiation environment, and thus analyzing all types of irradiated microstructures is not feasible. In the present study, fractal analysis is used for the suitability of structural representation in addressing the change in microstructure by irradiation energy. A more accurate relation for the fractal dimension of the irradiated W microstructures can be developed if more data from the microscopic studies performed in a variety of irradiation conditions are available. In order to develop the model with an account of realistic irradiation conditions, collaborative research is under way, and this will be reported in the subsequent paper in the future. 

Analyses in the present study show that the fractal dimension of the irradiated W microstructures decreases with an increase in irradiation energy. Because only a limited amount of data on irradiation experiments is now available, the empirical relationship proposed in this work was developed based on the fractal analysis largely on the W samples irradiated by low irradiation energy and over a short duration. However, it is worth noting that the correlation between the fractal dimension and irradiation energy was found for all examined W microstructures.

### 2.2. Phase-Field Model

This section presents a phase-field model to study the evolution of voids in W polycrystalline under He/D irradiation. The vacancy concentration, c(r,t) is chosen as a phase-field variable, which is continuous in space and evolves in time. The formation of voids occurs due to the condensation of vacancies and thus the void can be defined as a phase with a vacancy concentration of 100% (i.e., c(r,t) = 1). Following the Cahn–Hilliard definition of the free energy for a non-uniform system [15], the total free energy functional of the material system E is expressed in terms of the vacancy concentration c(r,t):

(3) $$E=\int [F(c(r,t),\;T)+\frac{\kappa }{2}{\left|\nabla c\left(r,t\right)\right|}^{2}+g(D)]\;dV,$$

where F(c(r,t), T) is the chemical free energy density (the details will be addressed later); *T* is the temperature; κ is the gradient energy coefficient associated with the void surface energy; *D* is the fractal dimension of W microstructure; and *g*(*D*) is the interfacial energy density induced by the crystallographic mismatch between grains.

**Figure 3:**
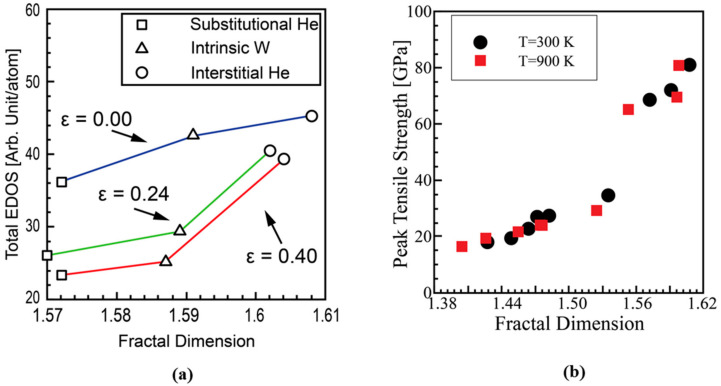
Ab initio simulation results of uniaxial tensile tests of W nanostructures reproduced from [22]. (**a**) Correlation between fractal dimension and total electron density of states (EDOS); (**b**) correlation between fractal dimension and peak tensile strength of W grain boundaries.

Han and Tomar [22] have used a fractal-dimension-based approach to correlate the changes in the atomic configuration of W grain boundaries (GBs) with the corresponding fractal dimensions. They reported that the bond strength of the examined W GBs is directly correlated with the fractal dimension of the corresponding W nanostructures as shown in Figure 3. Based on the ab initio simulation results, the relation used to predict the strength of W GBs was developed as a function of the fractal dimension and temperature. In the present work, such a relation is used for the interfacial energy density(g(D)) of the crystallographic mismatch between grains. For a better understanding of the variation of interfacial energy in polycrystalline, the atomic structure and configuration of the atomic bonding at the interface should be taken into account [38]. A key characteristic of the formulation in the present study is that the variation of the crystallographic mismatch energy at the interface can be taken into account as a function of He/D irradiation energy using Equation (2).

#### 2.2.1. Chemical Free Energy Density

In this work, the chemical free energy density in reference [9] is used for the binary system consisting of voids and the matrix:

(4) $$F(c,\;T)=k_{B}T[(1-\;c)\ln(1-\;c)+c\ln c]+(\;f_{0}c^{4}+f_{1}c^{3}+f_{2}c^{2}+f_{3}c+f_{4}),$$

**Figure 4:**
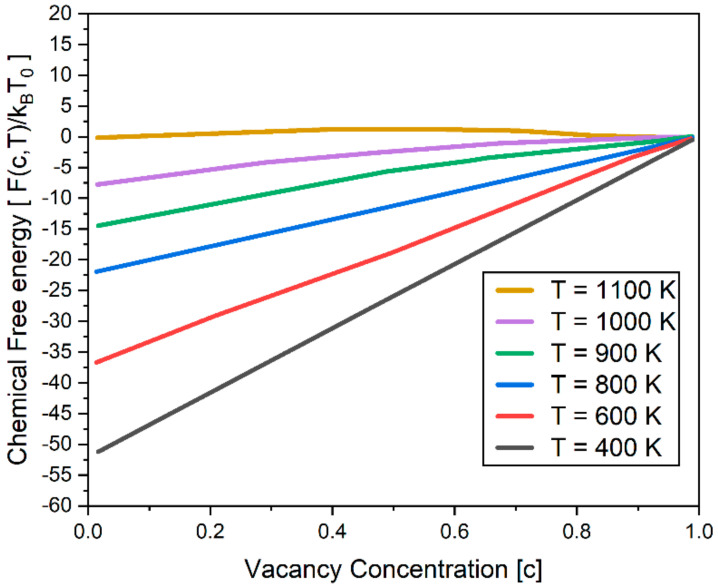
Chemical free energy used in the simulation. *T*_0_ = 1100 K is taken as the reference state where the free energies of void and matrix phases are zero.

The first term of Equation (4) represents the entropy contribution to the binary system with the Boltzmann constant, $k_{B}$. The second term is the enthalpy contribution of vacancy in the matrix. The coefficient $f_{i}$ (i = 0,1,2,3,4) depends on the temperature T. In order to describe the microstructure evolution accurately, the chemical free energy function is constructed such that it captures the following thermodynamic properties [9]: (1) The equilibrium vacancy concentration in the matrix decreases with decreasing temperature, while the equilibrium vacancy concentration for the void phase remains constant; (2) the free energy of the matrix phase decreases with decreasing temperature, while the free energy of the void phase is zero, independent of temperature. Figure 4 shows the chemical free energy used in the present work. In reference [9], the chemical free energy plot is presented in Figure 4. Based on the figure, the coefficient *f_i_*(*i* = 0,1,2,3,4) in Equation (4) was determined using the nonlinear least-squares method and listed in Table 2.

Hu and Heneager selected *T* = 1100 K as the reference temperature in their previous study [9,39] such that the free energy of the matrix phase decreases with decreasing temperature, while the free energy of the void phase is zero, independent of temperature. Such a selection was made based on the experimental and computational studies on the chemical free energy of the void phase [40,41,42].

#### 2.2.2. Numerical Implementation

The kinetic equation for the spatial and temporal evolution of vacancy concentration c(r,t) is defined using the Cahn–Hilliard equation [15] as follows:

(5) $$\frac{\partial c}{\partial t}=\nabla \cdot \left[M_{v}\nabla \frac{\delta E(c)}{\delta c}\right],$$

where *E*(c) is the total free energy functional defined in Equation (3). *M*_v_ is the vacancy mobility, which is defined as

(6) $$M_{v}=\frac{D_{v}c}{k_{B}T},$$

where *D*_v_ is the vacancy diffusivity. Liu and co-workers [43] calculated the vacancy diffusivities in tungsten as a function of temperature using ab initio simulations. These values are used for the present phase-field study and summarized in Table 3.

The numerical solution of Equation (5) provides the temporal evolution of the phase-field variable, which represents the vacancy concentration in the system. The domain for the simulation was discretized with uniform grids, which are 540 × 320 mesh points in x and y directions, respectively. Periodic boundary conditions were imposed in the simulation domain. The average of the vacancy concentration c(r,t) was assumed initially to be zero (i.e., < c(r,t)) > = 0). Thus, c(r,t) was initially set to a gaussian distributed random variable with zero mean and a standard variation of 0.001. The explicit finite difference method with forward Euler time-marching was used to solve Equation (5).

## 3. Results

In this work, an SEM image from Gupta et al. (Figure 3 in [44]) was used for the phase-field modeling to study the kinetics of void evolutions in W polycrystalline under He/D irradiation. They analyzed the SEM image of W sintered at 1475 °C presenting the different GB configurations in the polycrystalline structure. The image in the reference is the only one available in the scientific report that precisely shows the morphology of grains and GBs. In the present study, the W polycrystalline structure was modeled for simulation purposes based on the SEM image.

**Figure 5:**
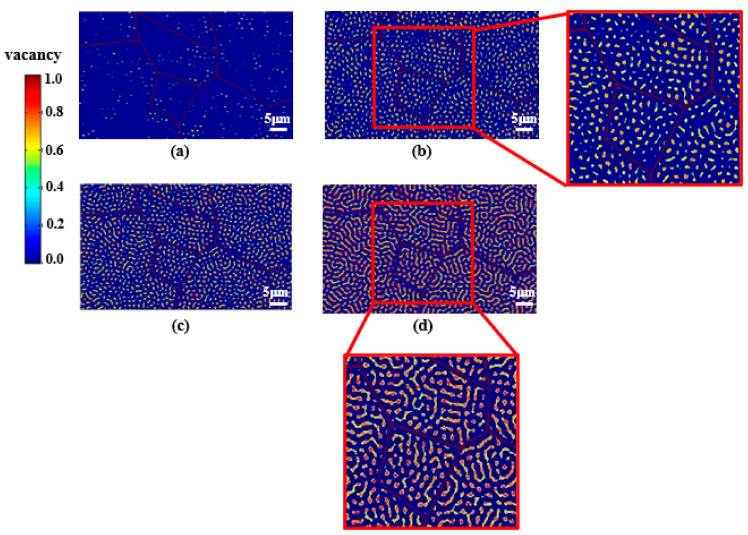
Temporal evolution of vacancy concentration in the W polycrystalline at T = 1000 K and E = 200 eV at the time of (**a**) t = 3.2 μs; (**b**) t = 4.4 μs; (**c**) t = 5.1 μs; (**d**) t = 6.8 μs.

Figure 5 shows the temporal evolution of the vacancy concentration (c(r,t)) in polycrystalline W at *T* = 1000 K and *E* = 200 eV. The regions contoured in red represent the voids where the vacancy concentration has a value of 1.0. In Figure 5a, vacancies start to form within grain interiors and a vacancy-depleted zone is observed along GBs at *t* = 3.2 μs. As time progress, the vacancy concentration increases, and a few voids are nucleated in grain interiors as shown in Figure 5b. The regions with the localized high vacancy concentrations can be considered as locations where a displacement cascade occurs at the early stage of the irradiation. At such an early stage, the increase in vacancy concentrations diffuses outward to the surrounding material rather than creating voids [45]. Such a process is also observed in Figure 5c showing that the density of vacancies increases throughout W polycrystalline. In Figure 5d, adjacent vacancies interact with each other and aggregate into vacancy clusters. These processes lead the morphology of vacancies to change from round to oval. The nucleation of voids due to the condensation of vacancies is also observed in Figure 5d. For the purpose of validation, the close-up views of Figure 5b,d can be compared with microscopic images of voids and vacancy clusters in W (from the references “Figure 3 in [24]” and “Figure 2 in [28]”, respectively). The comparison shows the qualitative similarities of void structures in terms of the oval shape of voids due to the aggregation and the relative size of the voids with respect to the grain size.

## 4. Discussion

**Figure 6:**
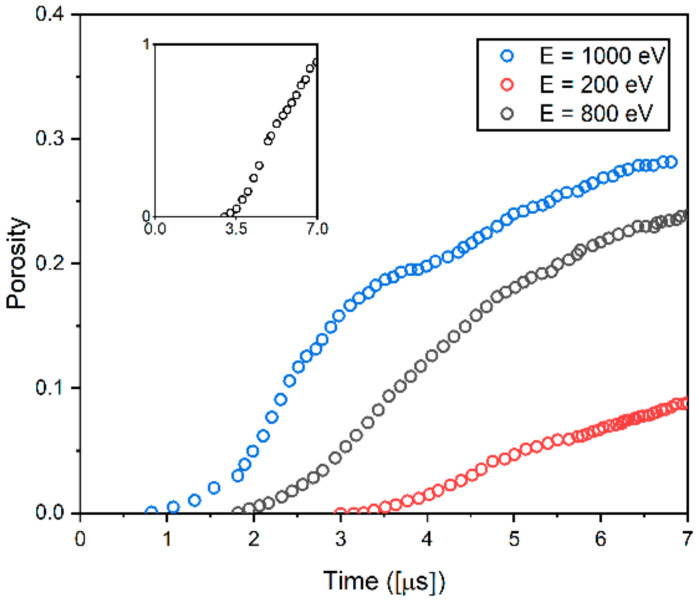
The evolution of porosity at T = 1000 K as a function of irradiation energy. Porosity is defined as a volume fraction of void.

The increase in the porosity due to the consecutive formation of voids under He/D irradiation leads to embrittlement, which significantly affects the material’s properties. Seeking insight into the influence of irradiation on porosity evolution, the temporal evolution of the porosity was calculated as a function of the irradiation energy. Porosity is defined as the percentage of void space in a simulation domain. Figure 6 shows the temporal evolution of porosity in the examined W polycrystalline for three different irradiation energies: *E* = 200 eV, 800 eV, 1000 eV. As shown in Figure 6, the incubation time for void nucleation increases with decreasing irradiation energy. While void phases start to form after the incubation time of *t* = 0.8 μs in the case of irradiation energy of *E* = 1000 eV, the incubation time increases to *t* = 3 μs for irradiation energy of *E* = 200 eV. As time progresses, voids start to be nucleated and grown. The competition between the surface energy and the chemical potential energy difference between solid and vacancy phases yields an energy barrier for void nucleation. Localized vacancies in the material system interact with each other and aggregate into vacancy clusters. If vacancy clusters grow large enough to overcome the free energy barrier for void nucleation, small stable voids are nucleated and grown [46]. In Figure 6, the trend of porosity evolution follows the Kolmogorov–Johnson–Mehl–Avrami (KJMA) equation. Transformation kinetics where nucleation and growth occur can be typically described by the Kolmogorov–Johnson–Mehl–Avrami (KJMA) equation [47,48,49], which is expressed as follows:

(7) $$X\left(t\right)=1-\exp\left(-k{\left(t-\tau \right)}^{m}\right)$$

where $X\left(t\right)$ is the volume fraction of the newly transformed phase, τ is the incubation time, *k* is the characteristic constant of nucleation kinetics in the system, and *m* is an exponent that characterizes the degree of heterogeneity of the system [46]. The KJMA equation has an S-curve form: That is, the nucleation rate is first accelerated and then decelerated, passing through an inflection point. Such a characteristic is also observed in Figure 6. Note that the inset of Figure 6 is plotted for the porosity evolution of irradiation energy, *E* = 200 eV, for better illustration. For all examined irradiation energies, after the incubation time is reached, the porosity increases exponentially during the void nucleation. Once the void nucleation is saturated, the porosity increase rate is found to be decreased. This is mainly because existing voids continue to grow by absorbing vacancies in the system, and the larger voids grow at the expense of the smaller ones [18]. Figure 6 also shows that in the case of the high irradiation energy, the nucleation-growth stage lasts longer than the one for the low irradiation energy.

When they are exposed to irradiation, microstructural features such as voids and He bubbles start to form. Localized vacancies in the microstructure interact with each other and aggregate into vacancy clusters. If vacancy clusters grow large enough to overcome the free energy barrier for void nucleation, small stable voids are nucleated and grown [25]. Once void nucleation is saturated, the porosity increase rate is found to be decreased as shown in Figure 6. The overall trend of Figure 2 is that the fractal dimension of the microstructures decreases with an increase in irradiation energy. It also shows that the sensitivity of the fractal dimension to the change in irradiation energy becomes small for the high level of the irradiation energy. In Figure 6, compared with the case of high irradiation exposure, the rate of void nucleation in the case of low irradiation energy is high. This indicates that the self-similarity (i.e., microstructure pattern) in the microscopic images for the low irradiation energy changes more sensitively than the one for the high irradiation energy. This can explain why the values of the fractal dimension decrease more sensitively at low irradiation than at high irradiation. Such a trend is also observed in another study. Jelčić et al. [50] performed a fractal analysis on the irradiated polymeric material(high impact polystyrene(PS-HI)). The results show that the fractal dimension of the irradiated polymer overall decreases with the increase in irradiation energy. They also suggest that the fractal dimension significantly decreases when increasing the irradiation energy and it is saturated in the case of high irradiation exposure, which is consistent with the present study. It is worth noting that the exponential function in Equation (2) was used for these reasons.

**Figure 7:**
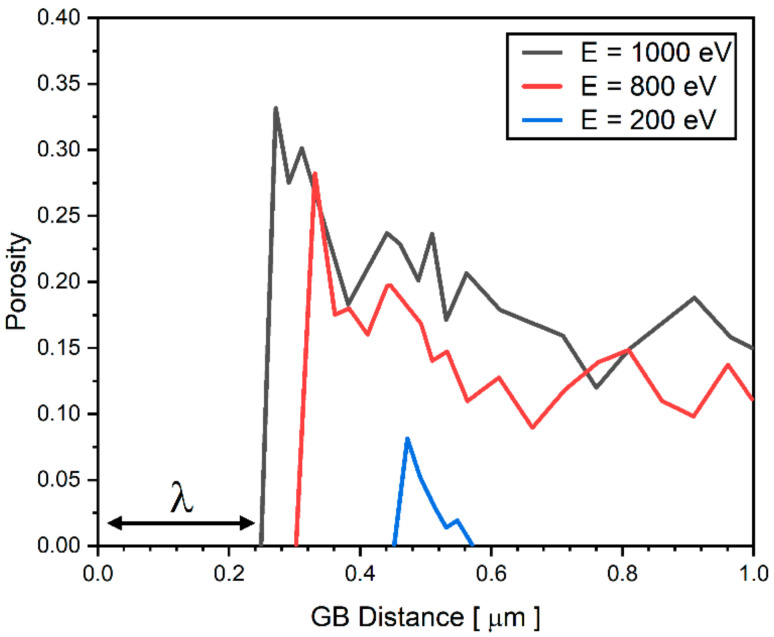
The porosity as a function of distance from GB for three different irradiation energies: E = 200 eV, E = 800 eV, and E = 1000 eV. The width of void denuded zone is marked as λ in the figure.

Grain Boundaries (GBs) are known as an efficient sink for the annihilation of irradiation-induced point defects and defect clusters [51,52]. As a result, the vacancy concentration near GBs is lower than the one in the interior of the grains. Because the vacancy concentration near GBs does not typically increase to the critical vacancy concentration for voids to nucleate and grow due to the decrease in supersaturation, the void-denuded zone (VDZ) forms near GBs. Figure 7 shows the porosity as a function of distance from GB for three different irradiation energies: *E* = 200 eV, 800 eV, 1000 eV. In Figure 7, the VDZ where porosity is zero is observed in the region near GB for all examined irradiation energies. As shown, porosity curves have a peak in the vicinity of the VDZ and decrease overall as it decreases from GBs. The width of VDZ (W_vdz_) is found to decrease with increasing irradiation energy. W_vdz_ for *E* = 200 eV is calculated as 0.44 μm while W_vdz_ for *E* = 800 eV and *E* = 1000 eV is calculated as 0.32 μm and 0.24 μm, respectively.

**Figure 8:**
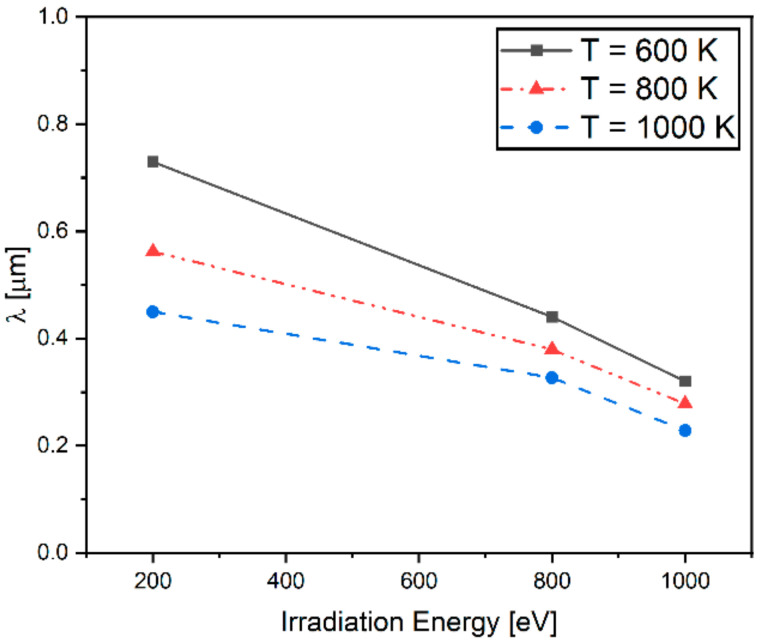
The effect of irradiation and temperature on the width of void denuded zone (λ).

In order to investigate the formation of VDZ under irradiation, the width of VDZs (W_vdz_) is plotted in Figure 8 as a function of irradiation energy and temperature. As shown, the W_vdz_ decreases with an increase in irradiation energy and temperature. It is also shown in Figure 8 that W_vdz_ changes more sensitively in response to the change in irradiation energy than the temperature change. The difference in the vacancy concentration between grain interiors and the vicinity of GBs leads to the diffusion of vacancies, and thus the formation of VDZs is controlled by such diffusion [52].

As shown in Figure 6, higher irradiation energy produces higher saturated vacancy and more voids, which result in the increase in the vacancy concentration gradient for a given polycrystalline structure. Thus, the VDZs are shifted toward the GBs with increasing irradiation energy, leading to the reduction of the width of VDZs.

Analyses performed in Figure 1 and Figure 2 show that for different types of final equilibrated microstructures after being exposed under irradiation, the fractal dimension and irradiation energy correlation can be uniquely defined. In this work, the interfacial energy induced by the crystallographic mismatch between grains under irradiation was taken into account using a fractal-dimension–based approach. It is worth noting that this interfacial energy term was added to the total free energy for the evolution of vacancy concentration at GB in W polycrystalline. In Figure 8, the difference of porosity depending on irradiation energy results from the variation of interfacial energy due to change in GB configuration. Findings regarding the decrease in the width of VDZs under the influence of GB characteristics have also been observed in the previous study. Han et al. [53] investigated the effect of the GB character on the formation of VDZs under irradiation. Their experiments show that VDZ widths are increased with the GB character, including both misorientation and GB plane orientation.

Fractal analysis in the present study is performed for the W microstructures irradiated by light ions (He/D) because the limited data are only available for cases of the neutron or the heavy ion irradiations, which provide the image with high enough resolution to allow for the fractal analysis. The type of particle for irradiation could affect the analysis in this work. In the present study, fractal analysis is used for the suitability of structural representation in addressing the change in microstructure by irradiation energy. A more accurate relation for the fractal dimension of the irradiated W microstructures can be developed if more data from the microscopic studies performed in a variety of irradiation conditions are available.

## 5. Conclusions

Overall, analyses in the present study show that the fractal dimensions of the irradiated W microstructures decrease with an increase in irradiation energy. Because a limited amount of data regarding irradiation-induced microstructure damage is available, the empirical relationship proposed in this work was developed based on the fractal analysis largely on the W samples irradiated by low irradiation energy. However, analyses show a direct correlation between the fractal dimension and irradiation energy for all examined W microstructures. In this respect, this work offers a unification of the range of microstructure-irradiation damage data reported in the literature. Based on the observed trend regarding the change in the fractal dimension of irradiated microstructures, an empirical relation was developed to predict the microstructural change under irradiation as a unique function of the irradiation energy. In order to study the evolution of voids in W polycrystalline under irradiation, the proposed relation was implemented in the phase-field model formulation with an account of the interfacial energy induced by the crystallographic mismatch between grains under irradiation. The simulation results demonstrate that the current phase-field model has the capability of capturing the evolution of the void under irradiation, including nucleation and the growth of voids, and the sink efficiency for vacancy annihilation in the vicinity of GB.

## Figures and Tables

**Table 1:** Information on the He/D irradiation conditions used for the fractal analysis in the present study.

Irradiation Ion Energy	Surface Temperature [K]	Fluence	Flux	Irradiation Time	Reference
10 eV	1600	2.3 × 10^26/^m^2^			[29]
10 eV	2600	0.9 × 10^27/^m^2^	1.2 × 10^23/^m^2^ s	7200 s	[24]
15 eV	1780			3600 s	[27]
15 eV	2200	1.0 × 10^25/^m^2^	8.3 × 10^22/^m^2^ s	1000 s	[25]
26 eV	1950	1.7 × 10^26/^m^2^	2.6 × 10^23/^m^2^ s	660 s	[28]
30 eV	2100	2.6 × 10^27/^m^2^	3.7 × 10^23/^m^2^ s	7200 s	[24]
45 eV	1273	3.0 × 10^26/^m^2^	0.7–3 × 10^24^/m^2^ s	1000 s	[33]
50 eV	1400	6.0 × 10^24/^m^2^		375 s	[26]
50 eV	1890	1.7 × 10^26/^m^2^	7.5 × 10^22/^m^2^ s	2300 s	[27]
60 eV	2300	1.5 × 10^26/^m^2^	2.6 × 10^23/^m^2^ s	600 s	[28]
64 eV	2050	0.5 × 10^26/^m^2^	0.6 × 10^23/^m^2^ s	720 s	[28]
80 eV	2100	3.3 × 10^26/^m^2^	9.2 × 10^22^/m^2^ s	3600 s	[27]
8 keV	300	2.6 × 10^19^/m^2^	2.6 × 10^17^/m^2^ s		[31]
8 keV	873	4.0 × 10^21^/m^2^	1.0 × 10^21^/m^2^ s		[30]
25 keV	1800	4.0 × 10^22^/m^2^	8.8 × 10^22^/m^2^ s		[32]
30 keV	1173	1.0 × 10^21/^m^2^			[34]

**Table 2:** The coefficient *f_i_*(*i* = 0,1,2,3,4) in Equation (4).

Temperature [K]	*f_0_*	*f_1_*	*f_2_*	*f_3_*	*f_4_*
400	0.002	0.213	−0.323	1.907	−1.783
600	−1.951	3.850	−2.347	2.381	−1.914
800	0	0.185	−0.278	1.627	−1.521
900	1.985	−4.172	2.284	1.067	−1.127
1000	−0.784	1.669	−1.766	1.548	−0.668
1100	1.400	−2.919	1.337	0.170	0.001

**Table 3:** The diffusivity of vacancies in W at a temperature range of 300 K to 1000 K.

T	300 K	400 K	500 K	600 K	700 K	800 K	900 K	1000 K
D_v_	3.46 × 10^−37^	3.41 × 10^−30^	5.01 × 10^−26^	3.11 × 10^−23^	3.08 × 10^−21^	9.95 × 10^−20^	1.42 × 10^−18^	1.21 × 10^−17^

## Data Availability

Not applicable.
